# The Chylomicron: Relationship to Atherosclerosis

**DOI:** 10.1155/2012/784536

**Published:** 2011-10-05

**Authors:** Gerald H. Tomkin, Daphne Owens

**Affiliations:** ^1^Diabetes Institute of Ireland, Beacon Clinic, Sandyford, Dublin 18, Ireland; ^2^Trinity College Dublin, Dublin 2, Ireland

## Abstract

The B-containing lipoproteins are the transporters of cholesterol, and the evidence suggests that the apo B48-containing postprandial chylomicron particles and the triglyceride-rich very low density lipoprotein (VLDL) particles play an important part in the development of the plaque both directly and indirectly by their impact on LDL composition. The ratio of dietary to synthesised cholesterol is variable but tightly regulated: hence intervention with diet at best reduces serum cholesterol by <20% andusually <10%. Statins are the mainstay of cholesterol reduction therapy, but they increase cholesterol absorption, an example of the relationship between synthesis and absorption. Inhibition of cholesterol absorption with Ezetimibe, an inhibitor of Niemann Pick C1-like 1 (NPC1-L1), the major regulator of cholesterol absorption, increases cholesterol synthesis and hence the value of adding an inhibitor of cholesterol absorption to an inhibitor of cholesterol synthesis. Apo B48, the structural protein of the chylomicron particle, is synthesised in abundance so that the release of these particles is dependent on the amount of cholesterol and triglyceride available in the intestine. This paper will discuss cholesterol absorption and synthesis, chylomicron formation, and the effect of postprandial lipoproteins on factors involved in atherosclerosis.

## 1. Introduction

The formation of the chylomicron is complex. In healthy humans virtually all fat is absorbed, but cholesterol is tightly regulated depending on body needs. A very flexible mechanism has evolved to keep serum cholesterol in a very narrow range. The chylomicron is responsible for the transport of medium- and long-chain fatty acids, together with cholesterol into the lymph. Apo B 48, the solubilising protein for the chylomicron, is secreted by the entrecote. Unused protein is degraded, a mechanism that ensures that there is sufficient apo B48 for even the largest fat meal. Fat feeding increases apo AIV expression, and apo A1V serves as a surface component for apo B48 particles in the entrecote [1, 2]. Apo A IV may stimulate net transfer of membrane triglyceride to luminal particles. It has been suggested that this occurs due to an increase in microsomal triglyceride transfer protein (MTP) at the pretranslational level [3]. The chylomicron particle comes in many sizes; thus the excessive fat load may be carried by an increase in particle numbers and/or size. Chylomicron assembly may involve 3 major processes: assembly of primordial lipoproteins, formation of lipid droplets, and core expansion [4].

## 2. The Chylomicron

The chylomicron is assembled mainly in the ER and is then transported to the *cis*-golgi in prechylomicron transport vesicles (PCVs) [5–7]. The secretion of the particles from the intestine is regulated by MTP. Among the functions of MTP is the ability to initiate the incorporation of lipids into apo B preventing apo B degradation. MTP acts as a chaperone to assist in apo B folding [8] (For excellent review of intestinal lipid absorption see Iqbal and Hussain [9]). Inhibitors of MTP in the intestine cause steatorrhoea are associated with weight loss and a reduction in cholesterol and triglycerides in human studies [10].

The chylomicron may be thought of as a particle that has as its main function the transport of fat from the intestinal lumen to the liver. On the way to uptake by the liver, the chylomicron divests itself of lipid to the adipocyte for storage or the muscle and other cells for energy. It also carries cholesterol which is made available following the controlled absorption under the regulation of NPC1L1 and ATP binding cassette proteins (ABC) G5/G8. Chylomicron cholesterol is also available from the enterohepatic circulation and from de novo synthesis by the enterocyte. To study the chylomicron particle formation it is interesting to examine the effects of insulin resistance, a condition associated with an increase in chylomicrons. The fructose-fed hamster is a model of diet-induced insulin resistance. Wong et al. [11] examined the proteomic profiles of prechylomicron transport vehicles (PCVs) isolated from the enteric endoplasmic reticulum in the small intestine. They found a number of PCV-associated proteins to be differentially expressed in these animals including MTP, apo B48, Sar-1, and vesicle-associated membrane protein 7** (**VAMP-7). Glucagon-like peptide-2 (GLP-2), a gastrointestinally derived intestinotropic hormone that links nutrient absorption to intestinal structure and function, was administered to hamsters and was found to increase secretion of apo B48, triglyceride-rich lipoprotein (TRL), and cholesterol mass. GLP 2 directly stimulated apo B48 secretion in jejunal fragments cultured ex vivo [12]. They further suggest that GLP2 represents a nutrient signal that regulates intestinal absorption of lipid and the assembly and secretion of chylomicrons from intestinal enterocytes. CD36 is a member of the class B scavenger receptor family of cell surface proteins, and the ability of GLP2 to increase intestinal lipoprotein production was lost in the CD36 −/− mice suggesting that the mechanism of action of GLP2 is through CD 36. 

To examine how cholesterol might influence the chylomicron it is interesting to consider the impact of plant sterols which lower intestinal absorption of cholesterol. Amiot et al. [13] have recently shown that plant sterol esters reduced meal-derived labeled cholesterol in chylomicrons but did not alter triglyceride hydrolysis or change chylomicron lipids nor the time course of postprandial chylomicron lipid increase. Ezetimibe inhibits NPC1L1 by binding to the protein and preventing conformational changes necessary for translocation of cholesterol across the membrane [14]. Bozzetto et al. [15] examined the role of Ezetimibe both fasting and after a standard meal in type 2 diabetic patients. They found that Ezetimibe added to a statin significantly decreased chylomicron cholesterol and triglyceride concentrations and postprandial apo B48. Masuda et al. [16] have shown that Ezetimibe inhibits postprandial hyperlipidaemia in patients with Type 2b hyperlipidaemia. CD 36 deficient mice have enhanced synthesis of chylomicrons in the small intestine. Sandoval et al. [17] showed that Ezetimibe reduces postprandial hyperlipidaemia in both wild-type and CD36 KO mice. They showed that triglyceride content and apo B48 mass were decreased and intestinal mucosal mRNA expression of fatty acid transfer protein 4 and apo B, along with fatty acid binding protein 2 (FAB2), diacylglycerol O-acyltransferase (DGAT)-1 and -2, and stearoyl-CoA desaturase (SCD)-1 which is involved in the synthesis and regulation of unsaturated fatty acids, were downregulated. It seems therefore that Ezetimibe has more actions than just down regulating cholesterol absorption, and these studies may help in evaluating the role of cholesterol in chylomicron assembly. It is particularly difficult to understand how triglyceride absorption is altered since Ezetimibe has not been shown to cause weight loss or steatorrhoea. Turnover studies might help to understand the role of NPC1-L1 in fat metabolism. It is possible that increased chylomicron particle clearance, due to the smaller load, plays a part in the above results.

## 3. Niemann Pick C1-Like1

The discovery of Niemann Pick C1-like1 (NPC1L1) is an interesting story. The search for an explanation as to how cholesterol in the body is so finely regulated has been intensive. The finely tuned regulation of cholesterol was perhaps best illustrated by the report of the elderly gentleman who ate 25 eggs a day for many years but his cholesterol remained at just above 6 mmol/L [18]. Altman and Davis in their search for molecules that might inhibit cholesterol absorption discovered by chance a compound which is now known as Ezetimibe [19]. They discovered a putative gene, the Niemann Pick C1-like1 (NPC1-L1). Elegant studies in mice demonstrated that knocking out this gene reduced cholesterol absorption by the same amount as happened when the wild mice were fed with Ezetimibe. They showed that there was no further reduction in cholesterol absorption in the knockout mice when fed Ezetimibe. The group went on to show that lack of NPC1L1 in apoE^−/−^ mice results in a significant reduction in cholesterol absorption and plasma cholesterol levels and caused a nearly complete protection from the development of atherosclerosis, under both cholesterol-fed and non-cholesterol-fed conditions [20, 21]. Weinglass et al. [14] have shown that Ezetimibe binds to a site distinct to the site where cholesterol binds, preventing conformational changes in NPC1L1 that is necessary for translocation of cholesterol across the membrane. Statins, which inhibit HMGCoA reductase and cholesterol synthesis, have been shown to increase cholesterol absorption. It has also been shown that low absorbers of cholesterol respond better to statins than high absorbers [22]. Ezetimibe potentiates the effect of statins, increasing their effectiveness by another 15–20% in relation to cholesterol lowering. Tremblay et al. [23] reported an increase in NPC1L1 by 19% on atorvastatin thus describing a mechanism whereby cholesterol absorption is increased in patients on statins. The mechanism of action of NPC1L1 has recently been further elucidated. It has been shown that cholesterol promotes the formation and endocytosis of NPC1L1 which appears to be an early step in cholesterol uptake. Zhang et al. [24] have discovered that it is the N-terminal domain of NPC1L1 that binds cholesterol. It is interesting that this domain does not bind to plant sterols; thus it now seems that plasma membrane-bound NPC21L1 binds exogenous cholesterol and this binding facilitates the formation of NPC1L1-flotiln-cholestrerol microdomains that are then internalized into cells through the clathrin AP2 pathway. In animal studies we have demonstrated an increase in cholesterol absorption in diabetes [25]; we asked the question as to whether diabetes might be associated with an increase in cholesterol absorption through stimulation of NPC1-L1. We demonstrated in animal models of diabetes that NPC1L1 was upregulated [26] and in diabetic patients, and we demonstrated an increase in NPC1L1 mRNA [27] suggesting a mechanism for an increase in cholesterol absorption. In the Psammomys Obesus, a model of type 2 diabetes, the animals exhibiting weight gain, hyperinsulinaemia, and hypercholesterolaemia, NPC1-L1 protein and gene expression were both significantly reduced in the intestine, and the authors found a lower capacity to absorb cholesterol compared to controls [28]. This may suggest interspecies variation but it is a surprising finding considering that this animal model of diabetes has been shown to have increased production of intestinal lipoprotein-containing apo B48 [29]. Ezetimibe has been shown to bind to the brush border and to NPCILI-expressing cells, [30]. There is a sterol regulatory element in the promoter and a sterol-sensing domain of NPCILI which appears to regulate cholesterol absorption in response to cholesterol intake. Huff et al. [31] have shown that NPC1L1 is suppressed in mice given a cholesterol-rich diet and increased in the cholesterol-depleted porcine intestine. The nuclear receptor, peroxisome proliferator-activated receptor (PPAR) delta/beta, appears to control the expression of NPC1L1. Activation by a synthetic agonist of PPAR delta has been shown to reduce cholesterol absorption and reduce expression of NPC1L1 without altering ABC G5/8 [32]. Tremblay et al. [23] have shown that Atorvastatin increases Niemann Pick C1L1 in the intestine and decreased ABC G 5/8 which leads to an increase in cholesterol absorption. These findings were accompanied by an increase in the transcription factors, sterol regulatory binding protein (SREBP) 2 and hepatic nuclear factor (HNF)-4. There may be other transporters of cholesterol, for example, scavenger receptor class B type 1 (SR-B1) [33] which is located both in the apical and basolateral membranes of the enterocyte. They may also play a role in cholesterol absorption. (For review see Iqbal and Hussein [9]). 

## 4. ATP Binding Cassette Proteins G5 and G8

The mechanism whereby the body is almost completely unable to absorb plant sterols was a mystery until recently. Study of the familial condition, sitosterolaemia, unlocked the mystery [34]. Sitosterolaemia is a rare condition associated with early and severe atherosclerosis. The condition is associated with normal or slightly elevated cholesterol whereas total sterols are markedly increased. Search for polymorphisms in putative genes, controlling plant sterol absorption or perhaps one should say blocking plant sterol absorption, identified ATP binding cassette proteins (ABC) G5 and G8 in the intestine [35]. Further work demonstrated that these two gene products work in tandem to reexcrete both plant sterols virtually completely and cholesterol to a lesser extent in a regulated way [36]. The genes were also found to be expressed in the liver where they are responsible for controlling cholesterol reexcretion into the bile [37]. It appears that these two genes are very important regulators of cholesterol and together with NPC1-L1 protein are responsible for cholesterol homeostasis in the body. 

Polymorphisms of the ABCG5/G8 have not only been associated with increased sitosterol but also with increase in cholesterol. It has also been shown that polymorphisms in the ABCG5/G8 may influence cholesterol in weight reduction programs (the Q604E SNP in ABCG5 and the C54Y in ABC8) [38]. Gylling et al. [39] examined polymorphisms in the ABCG5 and G8 genes and found that low serum cholesterol and cholesterol absorption were linked to a polymorphism (D19H) of the ABCG8 gene and characteristics of the insulin resistance syndrome in men were linked to Q604E polymorphism in the ABCG5 gene. The authors studied 263 mildly hypercholesterolaemic noncoronary subjects using cholestanol to cholesterol ratio as a surrogate marker of cholesterol absorption efficiency. 

Since diabetes is so frequently associated with dyslipidaemia and atherosclerosis, the ABCs became a target for research. Bloks et al. [40] examined mRNA and protein expression of ABCG5 and G8 in the intestine of streptozotosin diabetic rats and found significant reduction in expression of both ABCG5 and G8. They found that levels were partially normalised on insulin supplementation. We have shown that ABCG5 and G8 were reduced by more than 50% in the intestine of zucker diabetic fa/fa rats compared with lean rats although this did not reach statistical significance [41]. Insulin treatment caused a non-significant increase in ABCG5 and G8 mRNA. In another study of streptozotosin diabetic rats ABCG5 and G8 were both very significantly reduced in the intestine [26]. There was a negative correlation between ABCG5 and G8 and chylomicron cholesterol [26]. In the Psammomys Obesus, another model of diabetes, Levy et al. [28, 29] showed a reduction in ABC G5/G8 in the intestine. In the intestine of human subjects with type 2 diabetes, ABCG5 and G8 mRNA were both significantly lower compared to controls [27]. There was a negative correlation between ABCG5 and G8 and NCP1-L1 in the combined diabetic and control subjects [27]. There was a significant negative correlation between chylomicron cholesterol and both ABCG5 and G8 [27]. These two genes appear to play an important role in the dysregulation of cholesterol metabolism in diabetes.

As stated above inhibition of HMG CoA reductase with a statin has been shown to decrease ABC G5/8 as well as increasing NCP1L1 to increase cholesterol absorption [23]. In the Psammomys obesus animal model of type 2 diabetes the ABC G5 /8 reduction was associated with a reduction rather than increase in cholesterol absorption perhaps due to a reduction in NPC1L1 which the authors found, suggesting a difference between different animal models of diabetes [28]. 

Calcium appears to regulate lipids, at least in post menopausal women where supplementation has been shown to favorably alter lipids. Ma et al. [42] have shown in hamsters that improvement was associated with downregulation of NPC1L1 and MTP mRNA and upregulation of intestinal ABCG5/8.

## 5. Intestinal Microsomal Triglyceride Transfer Protein

Intestinal microsomal triglyceride transfer protein (MTP) plays a major role in the "assembly of the chylomicron particle and therefore of cholesterol and triglyceride metabolism. MTP has become a hot topic since inhibitors of intestinal MTP have been shown to lower triglyceride without causing hepatic steatosis at least in animal studies [43–45]. Although many polymorphisms of MTP have been described, some of which have considerable impact on LDL cholesterol in both nondiabetic and diabetic subjects [46, 47], it is difficult to know whether the results mainly stemmed from the effect in the liver rather than the intestine. The intestinal inhibitors of MTP which have no effect on the liver should answer this question in the future. In animal studies, diabetes is associated with an increase in MTP mRNA with close correlation between MTP mRNA and chylomicron cholesterol [40, 41, 48, 49]. In the rabbit increased intestinal MTP mRNA is associated with increase in chylomicron particle numbers [48], but in the rat it is associated with larger particles [49]. The fructose-fed insulin-resistant hamster model had an increase in MTP protein mass, and this was associated with an increase in the triglyceride-rich intestinally derived lipoproteins [50]. Zoltowska et al. in 2003 [51] examined the B48-containing lipoprotein assembly in the small intestine of *Psammomys obesus*, a model of nutritionally induced diabetes and insulin resistance. De novo triglyceride synthesis, apo B48 biogenesis, and triglyceride-rich lipoprotein assembly were all increased. MTP activity and protein expression, however, were not altered. In the enterocyte of fructose-fed golden hamster MTP mRNA and protein mass were increased by TNF*α*, but apo B levels in the enterocyte were not affected suggesting that there is considerable inter species variation [50]. In human studies in type 2 diabetes we demonstrated an increase in MTP mRNA in intestinal biopsies [27, 46]. Diabetic patients who were on statin therapy had lower MTP mRNA compared to those not on statins [46]. We found positive correlations between MTP mRNA and chylomicron fraction cholesterol and apo B48 [46].

## 6. MTP Polymorphism

In type 2 diabetes the common MTP 492 G/T polymorphism has been associated with surrogate markers of nonalcoholic hepatic steatosis [52]. The homozygous form of the polymorphism has been shown to be associated with a higher concentration of LDL 3 in diabetic patients of Chinese origin, but there was no effect in the heterozygous subjects [53]. In nondiabetic subjects heterozygous for the T allele no changes in LDL triglyceride or cholesterol have been found whereas the few subjects homozygous for the T allele had decreased numbers of triglyceride-rich VLDL particles and significantly lower VLDL and LDL cholesterol [54]. It has been suggested that the T allele might interact with visceral obesity and hyperinsulinaemia in nondiabetic subjects [55]. Examination of fasting lipids in a healthy black male population demonstrated that the rare T/T genotype was associated with a higher mean level of apo B suggesting a racial difference [56]. The effect of the influence of the G/T polymorphism on postprandial lipoproteins demonstrated that the TT polymorphism was associated with an increase in apo B48 in the smallest triglyceride-rich lipoprotein fraction postprandially without a difference in postprandial triglycerides or in fasting plasma or TRL cholesterol [54]. We have found in diabetic patients that the heterozygous −493 G/T polymorphism was associated with significantly lower LDL cholesterol and in the postprandial period higher apo B48 in the small chylomicrons [46]. It was a surprise to us that the −493 G/T homozygous subjects who had lower LDL cholesterol were reported to have an increase in CHD [57]. Homozygosity of the minor −493 T allele has been associated with an increased risk of IHD in 2 further studies [58, 59]. These intriguing findings have been further investigated by Aminoff et al. [60]. Aminoff et al. [60] showed that both the MTP polymorphisms −493 G > T and the 164 T > C result in lower transcription of MTP in vivo in the heart, liver, and macrophage. They showed in a case-controlled study that the subjects homozygous for −164 C allele had an increased risk of IHD. These studies, together with the knowledge that the heart secretes Apo B-containing lipoproteins, suggest that reduction in MTP in the heart results in lipid accumulation in the heart and is followed by IHD or the susceptibility to IHD perhaps through reduction in availability of free fatty acids for energy at times of acute stress. Of course another theory is that just like the liver the decrease in secretion of VLDL through reduction in MTP function that leads to hepatic steatosis in the heart leads to accumulation of fat that may be toxic to the myocardium. Last year Bharadwaj et al. [61] demonstrated that in an animal model VLDL and chylomicron lipids enter the heart through different pathways. A CD36 process appears to be important for VLDL lipoproteins and a non-CD36 for chylomicron-derived fatty acid uptake. They showed that lypolysis is involved in the uptake of core lipids from triglyceride-rich lipoproteins. AMPK plays a central role in energy homeostasis. In the heart it increases during ischaemia and is thought to be implicated in the pathophysiology of cardiovascular and metabolic disease. AMPK has been targeted as being of value in the production of new therapies for cardiac and metabolic disease. AMPK is insulin sensitive, and in diabetes a reduction in AMPK activity leads to a decrease in muscle glucose uptake thus shifting fuel from glucose to fat for cardiac myocyte function. It is therefore interesting to speculate that the −493 polymorphism might be particularly a disadvantage to patients with diabetes. A further recent link to the possible importance of MTP activity in the cardiac muscle in diabetes is the finding that the redox-sensitive transcription factor NF-E2-related factor 2 (NrF2) is suppressed by extracellular signaling-related kinase (ERK) leading to an increase in stress-induced insulin resistance in cardiac myocytes [62]. The authors also showed in the hearts of streptozotosin-induced diabetic mice downregulation of glucose utilization. These studies demonstrate the importance of the chylomicron and MTP in cardiac function/dysfunction and may account at least to some extent for the worse prognosis in those diabetic patients who have a MI.

## 7. Regulation of Chylomicron Synthesis

**Figure 1:**
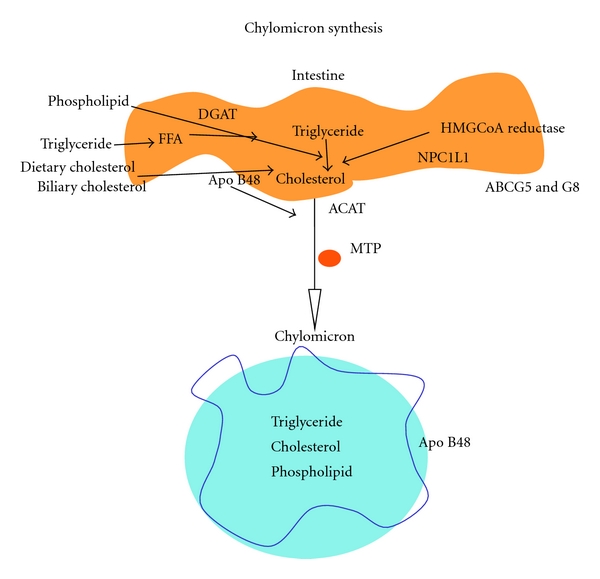
chylomicron synthesis. Dietary triglyceride, phospholipid, and cholesterol, together with intestinally synthesized cholesterol (for which 3-Hydroxy-3-methylglutaryl coenzyme A (HMG-CoA) reductase is the rate-limiting enzyme) and recycled biliary cholesterol together with intestinally derived ApoB48 are assembled under the influence of microsomal triglyceride transfer protein (MTP) to form the chylomicron. Prior to incorporation into the chylomicron, cholesterol is esterified by acylcoenzyme A:cholesterol acyltransferase (ACAT) and the triglycerides are reassembled from fatty acids with acylcoenzyme-A-diacylglycerol acyltransferase (DGAT) catalysing the rate-limiting step. The cholesterol is transferred across the membrane by Niemann Pick C1 like-1 and some is reexcreted back into the intestinal lumen by the action of ATP-binding cassette proteins (ABC)G5 and -G8. The particle is assembled under the regulation of microsomal triglyceride transfer protein and delivered to the lymphatics.

The synthesis of triglyceride in the liver depends on acylcoenzyme-A-diacylglycerol acyltransferase (DGAT). This enzyme is also found in many other tissues including the intestine and white adipose tissue, tissues that are active in triglyceride syntheis. Two DGAT enzymes have been discovered, DGAT1 and DGAT 2. These enzymes catalyse the final step of the triglyceride pathway [63], their substrates being diacyl glycerol and fatty acyl CoA. DGAT-1 deficient mice are resistant to diet-induced obesity and have increased sensitivity to insulin and leptin [64, 65], hence the excitment in discovering DGAT inhibitors [66, 67]. DGAT stimulates PPARs, and PPARa regulates lipid metabolism through its affect on adipocyte formation. Activation of PPARa with Fibrates lowers triglyceride through a number of mechanisms including increasing free fatty acid *β*-oxidation, hepatic lipoprotein lipase expression, a reduction in apo C111, and a reduction in apo B [68]. The mechanism by which PPAR*α* activation represses apoCIII transcription has yet to be elucidated. In vitro studies imply repression of apoCIII expression via interaction with a PPRE in the Rev-erb-*α* promoter, since it has been shown that mice deficient in this protein exhibit increased plasma concentrations of TG and apoCIII [69]. Whether apoCIII affects TG metabolism in vivo is contentious. Some studies showed an effect in normolipidemic subjects [70, 71]. Increases in the number and apo CIII content of VLDL particles also have adverse consequences for other lipoprotein subspecies, contributing to an increase in small, dense LDL particles [72]. The role of fenofibrate in the prevention of atherosclerosis is still disputed with large trials such as the field study[73] failing to demonstrate benefit for primary endpoints although secondary endpoints suggested that benefit and the DAIS study [74], an angiographic study in diabetes, certainly demonstrated significant reduction in the progression of atherosclerotic lesions (Figure 1).

The role of Apo A-IV is of interest as it increases MTP activity and leads to increased lipidation of the chylomicron particle (For review see Black [75]). In newborn swine, intestinal epithelial cells that had overexpressed apo A-IV increased the lipid content of the chylomicron particle [76, 77]. Further studies showed that the mechanism was through upregulation of MTP at the pretranslational level [78]. A meal rich in fat increases Apo A IV synthesis. During lipolysis of the chylomicron particle apo A IV binds to HDL although some circulates free. The function of apo AIV on HDL is not clear, but reduced levels are associated with cardiovascular disease, and transgenic overexpression protects mice fed a high-fat diet from atherosclerosis [79] suggesting that Apo A IV plays an important regulatory role in fat absorption and storage. Fasting jejunal lipid content was examined in morbidly obese persons some of whom had diabetes [80]. The diabetic subjects had lower triglyceride levels but Apo A IV mRNA was significantly higher with a significant negative correlation between apo IV mRNA expression and jejunal triglyceride. The diabetic patients had higher chylomicron triglycerides and apo B48. In the fructose-fed hamster, a model of insulin resistance, it has been demonstrated that the intestine is not responsive to the insulin-induced downregulation of the apo B48 lipoprotein production found in the chowfed animals [81]. The mechanism appears to be through disturbance in the ERK pathway which involves both insulin signaling and lipoprotein overproduction. These results are in keeping with the studies which have shown that insulin resistance and diabetes are associated with an increase in MTP in the liver and that MTP is negatively regulated by insulin [82–84]. In further studies reported by the Toronto group in 2010 [85] the prechylomicron transport vesicles (PCVs) were characterized, and proteomic profiles were developed. They have reported that MTP, Apo B48, SAR-1, and VAMP-7 were differentially expressed when compared to the chow fed animals. The results the authors suggest have increased our understanding of the assembly and transport of nascent chylomicrons in insulin-resistant states.

The intestinal enterocyte has a short half-life but yet manages to finely control fat absorption so that even the largest fat meal does not pass through unabsorbed. The mechanism involves increase in chylomicron number as shown by increased apo B and increased size as shown by the fat content of the chylomicron. Dai et al. [86] examined the mechanism whereby CaCo 2 cells differentiate into enterocyte cells and secrete chylomicron-like apo B48 particles when incubated with oleic acid, whereas cells that go on to become crypt cells do not have this ability. They found that MTP expression seemed to be a limiting factor for apo B lipoprotein secretion. They found evidence that HNF1, HNF4, and DR1 were critical for differentiation-dependent MTP induction and that repression was induced by NR2F1 and IRE1B. NR2F1 and IRE1B were found more in the crypts than in the villi suggesting the mechanism whereby only the enterocyte has the facility to absorb fat and that MTP is the limiting factor. These experiments add to the excitement of intestinal MTP as a target for treatment of dyslipidaemia in diabetes and other conditions where there is excess of postprandial chylomicrons.

## 8. Gene Regulation of Chylomicron Metabolism

A high-fat diet is associated with an increase in triglyceride-rich lipoproteins. Hernández-Vallejo et al. [87] investigated the impact of a short-term high-fat diet in mice and showed that apo B, MTP, and Apo A IV were upregulated to handle the increased lipid load. They also showed that there was a suppression of genes associated with fatty acid synthesis, FAS, ACC, SREBP-1c, and a key regulator of lipid biosynthesis was increased and translocated to the nucleus. LXR is considered to be a central player in energy homeostasis as indicated by its putative role in lipogenesis, gluconeogenesis, lipoprotein metabolism, and glucose uptake [88]. SREBP was only partly dependent on LXR. Apo A5 plays a major role in the metabolism of triglyceride-rich lipoproteins in the liver [89]. It is less certain whether apo A-5 plays a part in the chylomicron metabolis. Recently Guardiola et al. [90] have demonstrated gene expression mostly in the duodenum and colon. In vitro studies suggested that the protein may be functional, but this needs further investigation.

Obesity is associated with an increase in inflammatory cytokines such as TNF-a [91]. TNFa infusion has been shown to stimulate the overproduction of intestinal apo B48 as well as hepatically derived apo B 100 particles [92, 93]. TNF a increases MTP, and we found MTP to be increased in animal and human diabetes [26, 27, 48, 49], a condition that is associated with increased TNFa [94]. Recently Qin et al. [94] have shown that TNFa increased CD 36 which is, among other functions, an important fatty acid transporter. Thus a vicious cycle is put in place whereby excessive feeding increases chylomicron production which leads to insulin resistance through deposition of fat which in turn stimulates TNFa and other cytokines which increase insulin resistance which increases chylomicron production.

The delipidated chylomicron particle is cleared by the liver through the LDL receptor-related protein 1, (LDLR-related protein 1) receptor, and LDL B/E receptor. The LDLR is insulin sensitive, and the receptor is downregulated in insulin resistance [95]. Through a series of steps the lipid and cholesterol are repackaged and excreted by the liver as VLDL with apoB100 as the solubilising protein. The pathways in the liver are not dissimilar to those in the intestine, and like chylomicrons in the intestine, the VLDL particle will contain some de novo synthesised cholesterol. The liver like the intestine can regulate, at least to some extent, the amount of cholesterol in the VLDL particle by regulation of excretion of cholesterol through the bile. NPC1L1 plays a part in the liver in the regulation of cholesterol transport. Hepatic nuclear factor-1 (HNF-1) alpha and sterol regulatory element binding protein (SREBP)-2 appear to be important regulators of NPC1L1 in the liver [96]. It has also been shown that they have important binding sites within the human NPC1L1 promoter. The role of NPC1L1 in the liver is probably to divert cholesterol away from excretion in the bile [97]. A recent study in female Chinese women with gall stones has shown reduced NPC1l1 mRNA and protein in the liver and supersaturation of cholesterol in the bile [98]. Ezetimibe has not been shown to increase the risk of gall stones perhaps because the drug has its primary effect in reducing cholesterol absorption. Indeed in the golden Syrian hamster Ezetimibe reduced diet-induced increase in biliary cholesterol [99], and, in gallstone-susceptible mice fed lithogenic diets, Ezetimibe prevented gall stone formation [100]. Inhibition of NPC1L1 by Ezetimibe is associated with an improvement in hepatic steatosis. Jia et al. [101] have recently investigated the mechanism by deleting NPC1L1 in mice and inducing hepatic steatosis with a high fat diet. The knockout mice did not develop steatosis. Hepatic fatty acid synthesis and mRNA for genes regulating lipogenesis were reduced, and the knockout animals did not develop hyperinsulinaemia. Nomura et al. [102] demonstrated in Zucker rats that Ezetimibe improved hepatic insulin signaling as well as hepatic steatosis in both the liver and in cultured steatotic hepatocytes. The drug recovered insulin-induced Akt activation and reduced gluconeogenic genes. The relevance of this study to humans is not clear as patients with diabetes who are treated with Ezetimibe do not improve blood sugar control Kishimoto et al. [103].

## 9. Chylomicron Clearance

**Figure 2:**
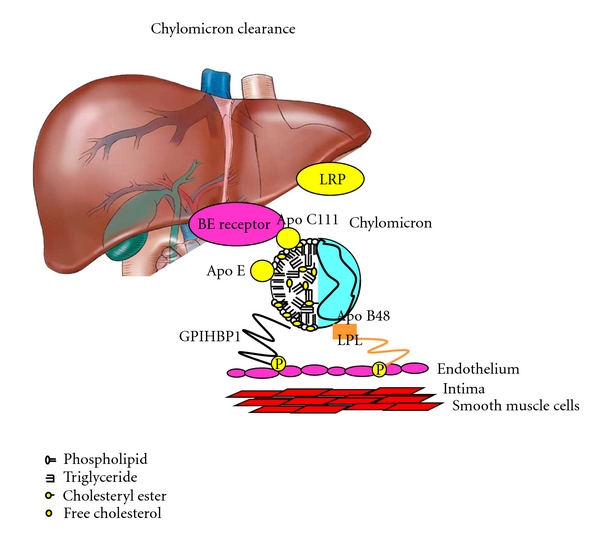
Chylomicron clearance. The chylomicron acquires apo E and apo C111 in the circulation, and most of the triglyceride is hydrolised by lipoprotein lipase (LPL) prior to it being cleared by the low density lipoprotein (LDL) B/E receptor or the LDL receptor-related protein (LRP) receptor in the liver. GPIHB also plays a smaller part in uptake of chylomicron remnants by the liver capillaries.

Delayed clearance of the chylomicron particle is another cause of hypertriglyceridaemia. The increased residence time of the particle potentially increases its atherogenicity and may be an important factor in the development of small dense LDL since large triglyceride-rich particles correlate well with small dense LDL. The delay in clearance of the chylomicron inevitably leads to a delay in clearance of VLDL since the apo BE receptor preferentially takes up the chylomicron particle. The triglyceride-rich lipoproteins may also be cleared in the liver by heparin sulfate proteoglycans HSPGs [104–106]. Among the HSPGs are the transmembrane syndecans, which have been shown to mediate the internalisation of model lipoproteins. Syndecan 1 can mediate binding and uptake of chylomicron remnants by HepG2 liver cells [107]. Stanford et al. [108] have shown this in in vivo genetic studies, using knockdown mice. Williams [104] showed that glycosylphosphatidyl inositol anchored high-density lipoprotein binding protein 1 (GPIHBP1) plays a critical role in the lypolytic processing of chylomicrons. GIHBP1 is located on the luminal face of the capillary endothelium and has been shown to bind both LpL and chylomicrons [109] suggesting that it serves as a platform for lipolysis drawing the chylomicron and LpL into close proximity. The role in atherosclerosis of this protein has still to be explored; however, a missense mutation of GIHBP1 in a young boy with severe chylomicronaemia has been described, and one presumes that other polymorphisms with less severe chylomicronaemia will help to unravel the role in atherosclerosis of this protein. Another cause for hypertriglyceridaemia is a mutation in HSPGs. Lipoprotein lipase (LpL) is stored in the subendothelial compartment awaiting transport. GPIHBp facilitates the transport of LpL from the subendothelium to the luminal side of the vasculature [110]. Bishop et al. [111] have demonstrated a deletion of the HPSG, Collagen 18, which resulted in reduced vascular LpL mass and activity in mice and caused mild hypertriglyceridaemia. Patients with collagen 18 deficiency have Knoblauch syndrome, a rare disorder characterised primarily by ocular defects. These patients have hypertriglyceridaemia (Figure 2) [111]. 

## 10. Chylomicron and Atherosclerosis

**Figure 3:**
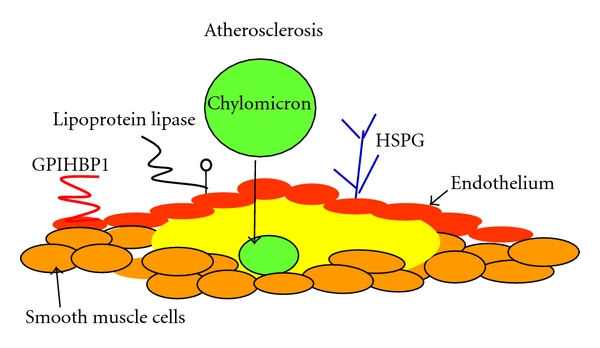
The chylomicron and atherosclerosis. The atherosclerotic plaque is composed of a lipid-rich core containing cholesterol and necrotic tissue and is covered by a fibrous smooth muscle cell cap. Low density lipoprotein (LDL) is the major contributor to plaque cholesterol, but chylomicron remnants are also taken up into the subendothelial space, and because of the rapid turnover of chylomicrons the amount of cholesterol they can deliver to the plaque is not reflected in serum chylomicron cholesterol. Chylomicrons are delipidated by lipoprotein lipase on the artery wall. They are attached to the endothelium by high-density lipoprotein-binding protein 1 (GPIHBP1) and heparin sulphate proteoglycans (HPSG) which facilitate their uptake into the subendothelial space. Chylomicron remnants become trapped in the artery wall and disintegrate to contribute cholesterol to the lipid-rich core.

Whereas cholesterol is tightly regulated with carefully evolved mechanisms to ensure that dietary cholesterol deficiency does not impede the body's need for cholesterol, the triglyceride metabolism is exquisitely regulated so that almost no dietary fat is lost, energy being conserved for later famine. The atherosclerotic plaque is mostly made up of cholesterol, fatty acids, and fibrous tissue. The recognition that, in conditions of severe hypercholesterolaemia such as familial hypercholesterolaemia and familial combined hyperlipidaemia, life expectancy was severely reduced due to atherosclerosis focused attention on cholesterol as being a major player in the atherosclerotic process. Population studies have confirmed the association. LDL cholesterol is the major cholesterol-containing particle. If cholesterol is measured per particle the LDL particle contains 100-fold more as compared to the chylomicron and the statins, which inhibit cholesterol synthesis and have been demonstrated to lower mostly LDL cholesterol and to reduce atherosclerosis. This has tended to focus attention even more strongly on the LDL particle as being the major player in atherosclerosis to the determent of the chylomicron. The chylomicron cholesterol content may be low, but the half-life is in minutes (rather than around 4 days for the LDL particle), hence the cholesterol carrying power of these particles is enormous and similar to LDL. More than 10 years ago Karpe et al. [112] demonstrated a relationship between apo B48 and carotid atherosclerosis both in normotriglyceridaemic and hypertriglyceridaemic subjects An argument that the chylomicron particle could not be considered as an atherogenic particle because of its size and therefore its inability to enter the subendothelial space is no longer valid since apo B48 has been found in atherosclerotic plaques in both animal and human studies [113, 114]. Proctor and Mamo [113] have demonstrated apo B48 in rabbit atherosclerotic plaque. The authors in elegant studies have also demonstrated that perfusing both chylomicron remnants and LDL in the rabbit aorta resulted in an preferential uptake of apo B48 in the subendothelial space suggesting that the chylomicron does indeed play a predominant role in the delivery of not only cholesterol but also of fatty acids to the plaque. Pal et al. in 2003 examined carotid endarterectomy patients and found apo B48 in the plaque [114]. These findings have been confirmed in humans by other workers [115]. The mechanism whereby the macrophage takes up the chylomicron particle has been extensively investigated. The chylomicron remnant competes for uptake of native LDL through the LDL receptor [116] but an apo B48-specific receptor has also been described [117, 118]. Elsegood et al. [119, 120] have described a 43 Kda macrophage chylomicron remnant binding protein as a candidate for sterol loading of macrophages resulting in the unabated uptake of chylomicron remnants by macrophages. It has been suggested that the chylomicron particle contains too little cholesterol to make it an important transporter of cholesterol to the atherosclerotic plaque but it must be remembered that the chylomicron particle number is massive in the postprandial phase compared to LDL and has a half-life in minutes rather than in days thus transporting over time, in relative terms, a similar amount of cholesterol compared to LDL. There have been as yet no clinical studies that have investigated the relationship between apo B48 and cardiovascular events, but it has been suggested that there is enough evidence to mount such trials [121]. Patients with type 1 diabetes as in type 2 diabetes are at increased risk of atherosclerosis. Mangat et al. [122] demonstrated increased apo B48 both fasting and postprandial in type 1 diabetic patients compared to controls. They also showed that the arterial retention of remnants ex vivo was increased 7-fold in type 1 diabetes relative to controls. The authors also showed that the remnants bound with significant affinity to human biglycan in vitro with a further 2-3-fold increase in binding activity with glycated glycan. The authors suggest that their findings support the hypothesis that impaired remnant metabolism may contribute to accelerated progression of atherosclerosis. We and others have shown increased Apo B48 in type 2 diabetes as compared to controls [123–126]. This abnormality was improved with better control of diabetes [123]. Taskinens group [126] has demonstrated an increase in apo B48 in diabetic patients with CVD as compared to diabetic patients without atherosclerosis. Finally it should be remembered that an increase in chylomicron cholesterol leads to and correlates with both VLDL cholesterol and LDL cholesterol, and large triglyceride-rich lipoproteins are associated with the atherogenic small dense LDL and low HDL. Both of these findings demonstrate the importance of the chylomicron in generating an atherogenic lipoprotein profile (Figure 3). 

In conclusion there is clear evidence that chylomicron metabolism is abnormal in diabetes, a condition which is associated with a heavy burden of atherosclerosis. There is good evidence to implicate the chylomicron directly in the atherosclerotic pathological process, and there is good evidence that abnormal chylomicron metabolism is associated with an atherogenic LDL profile. Measures to decrease chylomicron formation should decrease atherosclerotic burden, and the results of an intestinal MTP inhibitor are therefore awaited with great interest. Increasing chylomicron turnover may not prove useful since accelerated metabolism of the particle may just lead to increased deposition in the plaque and increased VLDL and an increase in small dense LDL. In both diabetic and nondiabetic subjects it seems wise to restrict chylomicron formation through strict diet and in diabetes; meticulous control of blood sugar will also improve chylomicronaemia. It is time to focus our attention on the chylomicron as an important player in the atherosclerotic process. The experimental animal studies and small human studies make it apparent that the time is right for large prospective clinical trials to evaluate the dangers of postprandial chylomicronaemia. With the advent of the specific intestinal inhibitors of MTP, it is likely that these studies will soon be undertaken. 

## References

[1] Hockey KJ, Anderson RA, Cook VR, Hantgan RR, Weinberg RB (2001). Effect of the apolipoprotein A-IV Q360H polymorphism on postprandial plasma triglyceride clearance. *Journal of Lipid Research*.

[2] Neeli I, Siddiqi SA, Siddiqi S (2007). Liver fatty acid-binding protein initiates budding of pre-chylomicron transport vesicles from intestinal endoplasmic reticulum. *Journal of Biological Chemistry*.

[3] Yao Y, Lu S, Huang Y (2011). Regulation of microsomal triglyceride transfer protein by apolipoprotein A-IV in newborn swine intestinal epithelial cells. *American Journal of Physiology*.

[4] Hussain MM, Kancha RK, Zhou Z, Luchoomun J, Zu H, Bakillah A (1996). Chylomicron assembly and catabolism: role of apolipoproteins and receptors. *Biochimica et Biophysica Acta*.

[5] Black DD (2007). Development and Physiological Regulation of Intestinal Lipid Absorption. I. Development of intestinal lipid absorption: Cellular events in chylomicron assembly and secretion. *American Journal of Physiology*.

[6] Kumar NS, Mansbach II CM (1999). Prechylomicron transport vesicle: isolation and partial characterization. *American Journal of Physiology*.

[7] Mansbach CM, Gorelick F (2007). Development and physiological regulation of intestinal lipid absorption. II. Dietary lipid absorption, complex lipid synthesis, and the intracellular packaging and secretion of chylomicrons. *American Journal of Physiology*.

[8] Jiang ZG, Liu Y, Hussain MM, Atkinson D, McKnight CJ (2008). Reconstituting initial events during the assembly of apolipoprotein B-containing lipoproteins in a cell-free system. *Journal of Molecular Biology*.

[9] Iqbal J, Hussain MM (2009). Intestinal lipid absorption. *American Journal of Physiology*.

[10] Tong W, Paradise E, Kim E (2010). Clinical investigations of SLx-4090 in combination with metformin in type 2 diabetes. *Diabetes Care*.

[11] Wong DM, Webb JP, Malinowski PM, Xu E, Macri J, Adeli K (2010). Proteomic profiling of intestinal prechylomicron transport vesicle (PCTV)-associated proteins in an animal model of insulin resistance (94 char). *Journal of Proteomics*.

[12] Hsieh J, Longuet C, Maida A (2009). Glucagon-Like Peptide-2 Increases Intestinal Lipid Absorption and Chylomicron Production via CD36. *Gastroenterology*.

[13] Amiot MJ, Knol D, Cardinault N (2011). Phytosterol ester processing in the small intestine: impact on cholesterol availability for absorption and chylomicron cholesterol incorporation in healthy humans. *Journal of Lipid Research*.

[14] Weinglass AB, Kohler M, Schulte U (2008). Extracellular loop C of NPC1L1 is important for binding to ezetimibe. *Proceedings of the National Academy of Sciences of the United States of America*.

[15] Bozzetto L, Annuzzi G, Corte GD (2011). Ezetimibe beneficially influences fasting and postprandial triglyceride-rich lipoproteins in type 2 diabetes. *Atherosclerosis*.

[16] Masuda D, Nakagawa-Toyama Y, Nakatani K (2009). Ezetimibe improves postprandial hyperlipidaemia in patients with type IIb hyperlipidaemia. *European Journal of Clinical Investigation*.

[17] Sandoval JC, Nakagawa-Toyama Y, Masuda D (2010). Molecular mechanisms of ezetimibe-induced attenuation of postprandial hypertriglyceridemia. *Journal of Atherosclerosis and Thrombosis*.

[18] Kern F (1991). Normal plasma cholesterol in an 88-year-old man who eats 25 eggs a day. Mechanisms of adaptation. *New England Journal of Medicine*.

[19] Altmann SW, Davis HR, Zhu LJ (2004). Niemann-Pick C1 Like 1 protein is critical for intestinal cholesterol absorption. *Science*.

[20] Davis HR, Hoos LM, Tetzloff G (2007). Deficiency of Niemann-Pick C1 Like 1 prevents atherosclerosis in apoE-/- mice. *Arteriosclerosis, Thrombosis, and Vascular Biology*.

[21] Davis HR, Lowe RS, Neff DR (2011). Effects of ezetimibe on atherosclerosis in preclinical models. *Atherosclerosis*.

[22] Lakoski SG, Xu F, Vega GL (2010). Indices of cholesterol metabolism and relative responsiveness to ezetimibe and simvastatin. *Journal of Clinical Endocrinology and Metabolism*.

[23] Tremblay AJ, Lamarche B, Lemelin V (2011). Atorvastatin increases intestinal expression of NPC1L1 in hyperlipidemic men. *Journal of Lipid Research*.

[24] Zhang J-H, Ge L, Qi W (2011). The N-terminal domain of NPC1L1 protein binds cholesterol and plays essential roles in cholesterol uptake. *Journal of Biological Chemistry*.

[25] Gleeson A, Owens D, Collins P, Johnson A, Tomkin GH (2000). The relationship between cholesterol absorption and intestinal cholesterol synthesis in the diabetic rat model. *Experimental Diabesity Research*.

[26] Lally S, Owens D, Tomkin GH (2007). Genes that affect cholesterol synthesis, cholesterol absorption, and chylomicron assembly: the relationship between the liver and intestine in control and streptozotosin diabetic rats. *Metabolism*.

[27] Lally S, Tan CY, Owens D, Tomkin GH (2006). Messenger RNA levels of genes involved in dysregulation of postprandial lipoproteins in type 2 diabetes: the role of Niemann-Pick C1-like 1, ATP-binding cassette, transporters G5 and G8, and of microsomal triglyceride transfer protein. *Diabetologia*.

[28] Levy E, Lalonde G, Delvin E (2010). Intestinal and hepatic cholesterol carriers in diabetic Psammomys obesus. *Endocrinology*.

[29] Levy E, Spahis S, Ziv E (2006). Overproduction of intestinal lipoprotein containing apolipoprotein B-48 in Psammomys obesus: impact of dietary n-3 fatty acids. *Diabetologia*.

[30] Garcia-Calvo M, Lisnock J, Bull HG (2005). The target of ezetimibe is Niemann-Pick C1-Like 1 (NPC1L1). *Proceedings of the National Academy of Sciences of the United States of America*.

[31] Huff MW, Pollex RL, Hegele RA (2006). NPC1L1: evolution from pharmacological target to physiological sterol transporter. *Arteriosclerosis, Thrombosis, and Vascular Biology*.

[32] Van Der Veen JN, Kruit JK, Havinga R (2005). Reduced cholesterol absorption upon PPAR*δ* activation coincides with decreased intestinal expression of NPC1L1. *Journal of Lipid Research*.

[33] Ashraf MZ, Gupta N (2011). Scavenger receptors: implications in atherothrombotic disorders. *International Journal of Biochemistry and Cell Biology*.

[34] Berge KE, Tian H, Graf GA (2000). Accumulation of dietary cholesterol in sitosterolemia caused by mutations in adjacent ABC transporters. *Science*.

[35] Graf GA, Yu L, Li WP (2003). ABCG5 and ABCG8 are obligate heterodimers for protein trafficking and biliary excretion. *Journal of Biological Chemistry*.

[36] Kosters A, Kunne C, Looije N, Patel SB, Oude Elferink RPJ, Groen AK (2006). The mechanism of ABCG5/ABCG8 in biliary cholesterol secretion in mice. *Journal of Lipid Research*.

[37] Wang HH, Patel SB, Carey MC, Wang DQH (2007). Quantifying anomalous intestinal sterol uptake, lymphatic transport, and biliary secretion in Abcg8-/- mice. *Hepatology*.

[38] Santosa S, Demonty I, Lichtenstein AH, Ordovas JM, Jones PJH (2007). Single nucleotide polymorphisms in ABCG5 and ABCG8 are associated with changes in cholesterol metabolism during weight loss. *Journal of Lipid Research*.

[39] Gylling H, Hallikainen M, Pihlajamäki J (2004). Polymorphisms in the ABCG5 and ABCG8 genes associate with cholesterol absorption and insulin sensitivity. *Journal of Lipid Research*.

[40] Bloks VW, Bakker-Van Waarde WM, Verkade HJ (2004). Down-regulation of hepatic and intestinal Abcg5 and Abcg8 expression associated with altered sterol fluxes in rats with streptozotocin-induced diabetes. *Diabetologia*.

[41] Lally S, Owens D, Tomkin GH (2007). The different effect of pioglitazone as compared to insulin on expression of hepatic and intestinal genes regulating post-prandial lipoproteins in diabetes. *Atherosclerosis*.

[42] Ma KY, Yang N, Jiao R (2011). Dietary calcium decreases plasma cholesterol by down-regulation of intestinal Niemann-Pick C1 like 1 and microsomal triacylglycerol transport protein and up-regulation of CYP7A1 and ABCG 5/8 in hamsters. *Molecular Nutrition and Food Research*.

[43] Kim E, Campbell S, Schueller O (2011). A small-molecule inhibitor of enterocytic microsomal triglyceride transfer protein, SLx-4090: biochemical, pharmacodynamic, pharmacokinetic, and safety profile. *Journal of Pharmacology and Experimental Therapeutics*.

[44] Hata T, Mera Y, Ishii Y (2011). JTT-130, a novel intestine-specific inhibitor of microsomal triglyceride transfer protein, suppresses food intake and gastric emptying with the elevation of plasma peptide YY and glucagon-like peptide-1 in a dietary fat-dependent manner. *Journal of Pharmacology and Experimental Therapeutics*.

[45] Aggarwal D, West KL, Zern TL, Shrestha S, Vergara-Jimenez M, Fernandez ML (2005). JTT-130, a microsomal triglyceride transfer protein (MTP) inhibitor lowers plasma triglycerides and LDL cholesterol concentrations without increasing hepatic triglycerides in guinea pigs. *BMC Cardiovascular Disorders*.

[46] Phillips C, Mullan K, Owens D, Tomkin GH (2004). Microsomal triglyceride transfer protein polymorphisms and lipoprotein levels in type 2 diabetes. *QJM*.

[47] Karpe F, Lundahl B, Ehrenborg E, Eriksson P, Hamsten A (1998). A common functional polymorphism in the promoter region of the microsomal triglyceride transfer protein gene influences plasma LDL levels. *Arteriosclerosis, Thrombosis, and Vascular Biology*.

[48] Phillips C, Bennett A, Anderton K (2002). Intestinal rather than hepatic microsomal triglyceride transfer protein as a cause of postprandial dyslipidemia in diabetes. *Metabolism*.

[49] Gleeson A, Anderton K, Owens D (1999). The role of microsomal triglyceride transfer protein and dietary cholesterol in chylomicron production in diabetes. *Diabetologia*.

[50] Qin B, Qiu W, Avramoglu RK, Adeli K (2007). Tumor necrosis factor-*α* induces intestinal insulin resistance and stimulates the overproduction of intestinal apolipoprotein b48-containing lipoproteins. *Diabetes*.

[51] Zoltowska M, Ziv E, Delvin E (2003). Cellular aspects of intestinal lipoprotein assembly in Psammomys obesus: a model of insulin resistance and type 2 diabetes. *Diabetes*.

[52] Bernard S, Touzet S, Personne I (2000). Association between microsomal triglyceride transfer protein gene polymorphism and the biological features of liver steatosis in patients with Type II diabetes. *Diabetologia*.

[53] Chen SPL, Tan KCB, Lam KSL (2003). Effect of the microsomal triglyceride transfer protein -493 G/T polymorphism and type 2 diabetes mellitus on LDL subfractions. *Atherosclerosis*.

[54] Karpe F, Lundahl B, Ehrenborg E, Eriksson P, Hamsten A (1998). A common functional polymorphism in the promoter region of the microsomal triglyceride transfer protein gene influences plasma LDL levels. *Arteriosclerosis, Thrombosis, and Vascular Biology*.

[55] St-Pierre J, Lemieux I, Miller-Felix I (2002). Visceral obesity and hyperinsulinemia modulate the impact of the microsomal triglyceride transfer protein -493G/T polymorphism on plasma lipoprotein levels in men. *Atherosclerosis*.

[56] Juo SHH, Han Z, Smith JD, Colangelo L, Liu K (2000). Common polymorphism in promoter of microsomal triglyceride transfer protein gene influences cholesterol, ApoB, and triglyceride levels in young African American men: Results from the coronary artery risk development in young adults (CARDIA) study. *Arteriosclerosis, Thrombosis, and Vascular Biology*.

[57] Ledmyr H, McMahon AD, Ehrenborg E (2004). The microsomal triglyceride transfer protein gene-493T variant lowers cholesterol but increases the risk of coronary heart disease. *Circulation*.

[58] Shepherd J, Cobbe SM, Ford I (1995). Prevention of coronary heart disease with pravastatin in men with hypercholesterolemia. *New England Journal of Medicine*.

[59] Lithell H, Aberg H, Selinus I, Hedstrand H (1984). The Primary Preventive Study in Uppsala: fatal and non-fatal myocardial infarction during a 10-year follow-up of a middle-aged male population with treatment of high-risk individuals. *Acta Medica Scandinavica*.

[60] Aminoff A, Ledmyr H, Thulin P (2010). Allele-specific regulation of MTTP expression influences the risk of ischemic heart disease. *Journal of Lipid Research*.

[61] Bharadwaj KG, Hiyama Y, Hu Y (2010). Chylomicron- and VLDL-derived lipids enter the heart through different pathways: in vivo evidence for receptor- and non-receptor-mediated fatty acid uptake. *Journal of Biological Chemistry*.

[62] Tan Y, Ichikawa T, Li J (2011). Diabetic downregulation of Nrf2 activity via ERK contributes to oxidative stress-induced insulin resistance in cardiac cells in vitro and in vivo. *Diabetes*.

[63] Chen HC, Farese RV (2000). DGAT and triglyceride synthesis: a new target for obesity treatment?. *Trends in Cardiovascular Medicine*.

[64] Smith SJ, Cases S, Jensen DR (2000). Obesity resistance and multiple mechanisms of triglyceride synthesis in mice lacking Dgat. *Nature Genetics*.

[65] Chen HC, Smith SJ, Ladha Z (2002). Increased insulin and leptin sensitivity in mice lacking acyl CoA:diacylglycerol acyltransferase 1. *Journal of Clinical Investigation*.

[66] Yamamoto T, Yamaguchi H, Miki H (2011). A novel coenzyme A:diacylglycerol acyltransferase 1 inhibitor stimulates lipid metabolism in muscle and lowers weight in animal models of obesity.

[67] Nakada Y, Ogino M, Asano K (2010). Novel acyl coenzyme A: dacylglycerol acyltransferase 1 inhibitors—synthesis and biological activities of N-(substituted heteroaryl)-4-(substituted phenyl)-4-oxobutanamides. *Chemical and Pharmaceutical Bulletin*.

[68] Schoonjans K, Peinado-Onsurbe J, Lefebvre AM (1996). PPAR*α* and PPAR*γ* activators direct a distinct tissue-specific transcriptional response via a PPRE in the lipoprotein lipase gene. *EMBO Journal*.

[69] Raspé E, Duez H, Mansén A (2002). Identification of Rev-erb*α* as a physiological repressor of apoC-III gene transcription. *Journal of Lipid Research*.

[70] Staels B, Vu-Dac N, Kosykh VA (1995). Fibrates downregulate apolipoprotein C-III expression independent of induction of peroxisomal acyl coenzyme A oxidase. A potential mechanism for the hypolipidemic action of fibrates. *Journal of Clinical Investigation*.

[71] Hiukka A, Fruchart-Najib J, Leinonen E, Hilden H, Fruchart JC, Taskinen MR (2005). Alterations of lipids and apolipoprotein CIII in very low density lipoprotein subspecies in type 2 diabetes. *Diabetologia*.

[72] Fruchart JC (2009). Peroxisome proliferator-activated receptor-alpha (PPAR*α*): at the crossroads of obesity, diabetes and cardiovascular disease. *Atherosclerosis*.

[73] FIELD Study Investigators (2005). Effects of long-term fenofibrate therapy on cardiovascular events in 9795 people with type 2 diabetes mellitus (the FIELD study): randomised controlled trial. *Lancet*.

[74] Steiner G, Hamsten A, Hosking J (2001). Effect of fenofibrate on progression of coronary-artery disease in type 2 diabetes: the Diabetes Atherosclerosis Intervention Study, a randomised study. *Lancet*.

[75] Black DD (2007). Development and physiological regulation of intestinal lipid absorption. I. Development of intestinal lipid absorption: cellular events in chylomicron assembly and secretion. *American Journal of Physiology*.

[76] Leng S, Lu S, Yao Y (2007). Hepatocyte nuclear factor-4 mediates apolipoprotein A-IV transcriptional regulation by fatty acid in newborn swine enterocytes. *American Journal of Physiology*.

[77] Lu S, Yao Y, Cheng X (2006). Overexpression of apolipoprotein A-IV enhances lipid secretion in IPEC-1 cells by increasing chylomicron size. *Journal of Biological Chemistry*.

[78] Yao Y, Lu S, Huang Y (2011). Regulation of microsomal triglyceride transfer protein by apolipoprotein A-IV in newborn swine intestinal epithelial cells. *American Journal of Physiology*.

[79] Cohen RD, Castellani LW, Qiao JH, Van Lenten BJ, Lusis AJ, Reue K (1997). Reduced aortic lesions and elevated high density lipoprotein levels in transgenic mice overexpressing mouse apolipoprotein A-IV. *Journal of Clinical Investigation*.

[80] Soriguer F, García-Serrano S, Garrido-Sánchez L (2010). Jejunal wall triglyceride concentration of morbidly obese persons is lower in those with type 2 diabetes mellitus. *Journal of Lipid Research*.

[81] Federico LM, Naples M, Taylor D, Adeli K (2006). Intestinal insulin resistance and aberrant production of apolipoprotein B48 lipoproteins in an animal model of insulin resistance and metabolic dyslipidemia: Evidence for activation of protein tyrosine phosphatase-1B, extracellular signal-related kinase, and sterol regulatory element-binding protein-1c in the fructose-fed hamster intestine. *Diabetes*.

[82] Au WS, Kung HF, Lin MC (2003). Regulation of microsomal triglyceride transfer protein gene by insulin in HepG2 cells: roles of MAPKerk and MAPKp38. *Diabetes*.

[83] Hagan DL, Kienzle B, Jamil H, Hariharan N (1994). Transcriptional regulation of human and hamster microsomal triglyceride transfer protein genes. Cell type specific expression and response to metabolic regulators. *Journal of Biological Chemistry*.

[84] Lin MCM, Gordon D, Wetterau JR (1995). Microsomal triglyceride transfer protein (MTP) regulation in HepG2 cells: insulin negatively regulates MTP gene expression. *Journal of Lipid Research*.

[85] Wong DM, Webb JP, Malinowski PM, Xu E, Macri J, Adeli K (2010). Proteomic profiling of intestinal prechylomicron transport vesicle (PCTV)-associated proteins in an animal model of insulin resistance (94 char). *Journal of Proteomics*.

[86] Dai K, Khatun I, Hussain MM (2010). NR2F1 and IRE1*β* suppress microsomal triglyceride transfer protein expression and lipoprotein assembly in undifferentiated intestinal epithelial cells. *Arteriosclerosis, Thrombosis, and Vascular Biology*.

[87] Hernández-Vallejo SJ, Alqub M, Luquet S (2009). Short-term adaptation of postprandial lipoprotein secretion and intestinal gene expression to a high-fat diet. *American Journal of Physiology*.

[88] Steffensen KR, Gustafsson JÅ (2004). Putative metabolic effects of the liver X receptor (LXR). *Diabetes*.

[89] Talmud PJ (2007). Rare APOA5 mutations–clinical consequences, metabolic and functional effects: an ENID review. *Atherosclerosis*.

[90] Guardiola M, Alvaro A, Vallvé JC APOA5 gene expression in the human intestinal tissue and its response to in vitro exposure to fatty acid and fibrate.

[91] Dandona P, Weinstock R, Thusu K, Abdel-Rahman E, Aljada A, Wadden T (1998). Tumor necrosis factor-*α* in sera of obese patients: fall with weight loss. *Journal of Clinical Endocrinology and Metabolism*.

[92] Qin B, Qiu W, Avramoglu RK, Adeli K (2007). Tumor necrosis factor-*α* induces intestinal insulin resistance and stimulates the overproduction of intestinal apolipoprotein b48-containing lipoproteins. *Diabetes*.

[93] Qin B, Anderson RA, Adeli K (2008). Tumor necrosis factor-*α* directly stimulates the overproduction of hepatic apolipoprotein B100-containing VLDL via impairment of hepatic insulin signaling. *American Journal of Physiology*.

[94] Qin B, Dawson H, Anderson RA (2010). Elevation of tumor necrosis factor-*α* induces the overproduction of postprandial intestinal apolipoprotein B48-containing very low-density lipoprotein particles: evidence for related gene expression of inflammatory, insulin and lipoprotein signaling in enterocytes. *Experimental Biology and Medicine*.

[95] Laatsch A, Merkel M, Talmud PJ, Grewal T, Beisiegel U, Heeren J (2009). Insulin stimulates hepatic low density lipoprotein receptor-related protein 1 (LRP1) to increase postprandial lipoprotein clearance. *Atherosclerosis*.

[96] Pramfalk C, Jiang ZY, Cai Q (2010). HNF1*α* and SREBP2 are important regulators of NPC1L1 in human liver. *Journal of Lipid Research*.

[97] Jia L, Betters JL, Yu L (2011). Niemann-Pick C1-Like 1 (NPC1L1) protein in intestinal and hepatic cholesterol transport. *Annual Review of Physiology*.

[98] Cui W, Jiang ZY, Cai Q (2010). Decreased NPC1L1 expression in the liver from Chinese female gallstone patients. *Lipids in Health and Disease*.

[99] Valasek MA, Repa JJ, Quan G, Dietschy JM, Turley SD (2008). Inhibiting intestinal NPC1L1 activity prevents diet-induced increase in biliary cholesterol in Golden Syrian hamsters. *American Journal of Physiology*.

[100] Zúñiga S, Molina H, Azocar L (2008). Ezetimibe prevents cholesterol gallstone formation in mice. *Liver International*.

[101] Jia L, Ma Y, Rong S (2010). Niemann-pick C1-like 1 deletion in mice prevents high-fat diet-induced fatty liver by reducing lipogenesis. *Journal of Lipid Research*.

[102] Nomura M, Ishii H, Kawakami A, Yoshida M (2009). Inhibition of hepatic Niemann-Pick C1-like 1 improves hepatic insulin resistance. *American Journal of Physiology*.

[103] Kishimoto M, Sugiyama T, Osame K, Takarabe D, Okamoto M, Noda M (2011). Efficacy of ezetimibe as monotherapy or combination therapy in hypercholesterolemic patients with and without diabetes. *Journal of Medical Investigation*.

[104] Williams KJ (2008). Molecular processes that handle—and mishandle—dietary lipids. *Journal of Clinical Investigation*.

[105] Mahley RW, Huang Y (2007). Atherogenic remnant lipoproteins: role for proteoglycans in trapping, transferring, and internalizing. *Journal of Clinical Investigation*.

[106] Mahley RW, Ji ZS (1999). Remnant lipoprotein metabolism: key pathways involving cell-surface heparan sulfate proteoglycans and apolipoprotein E. *Journal of Lipid Research*.

[107] Zeng BJ, Mortimer BC, Martins IJ, Seydel U, Redgrave TG (1998). Chylomicron remnant uptake is regulated by the expression and function of heparan sulfate proteoglycan in hepatocytes. *Journal of Lipid Research*.

[108] Stanford KI, Bishop JR, Foley EM (2009). Syndecan-1 is the primary heparan sulfate proteoglycan mediating hepatic clearance of triglyceride-rich lipoproteins in mice. *Journal of Clinical Investigation*.

[109] Beigneux AP, Davies BSJ, Gin P (2007). Glycosylphosphatidylinositol-anchored high-density lipoprotein-binding protein 1 plays a critical role in the lipolytic processing of chylomicrons. *Cell Metabolism*.

[110] Davies BSJ, Beigneux AP, Barnes RH (2010). GPIHBP1 is responsible for the entry of lipoprotein lipase into capillaries. *Cell Metabolism*.

[111] Bishop JR, Passos-Bueno MR, Fong L (2010). Deletion of the basement membrane heparan sulfate proteoglycan type XVIII collagen causes hypertriglyceridemia in mice and humans. *PLoS One*.

[112] Karpe F, Steiner G, Uffelman K, Olivecrona T, Hamsten A (1994). Postprandial lipoproteins and progression of coronary atherosclerosis. *Atherosclerosis*.

[113] Proctor SD, Mamo JCL (2003). Intimal retention of cholesterol derived from apolipoprotein B100- and apolipoprotein B48-containing lipoproteins in carotid arteries of Watanabe heritable hyperlipidemic rabbits. *Arteriosclerosis, Thrombosis, and Vascular Biology*.

[114] Pal S, Semorine K, Watts GF, Mamo J (2003). Identification of lipoproteins of intestinal origin in human atherosclerotic plaque. *Clinical Chemistry and Laboratory Medicine*.

[115] Nakano T, Nakajima K, Niimi M (2008). Detection of apolipoproteins B-48 and B-100 carrying particles in lipoprotein fractions extracted from human aortic atherosclerotic plaques in sudden cardiac death cases. *Clinica Chimica Acta*.

[116] Floren CH, Chait A (1981). Uptake of chylomicron remnants by the native LDL receptor in human monocyte-derived macrophages. *Biochimica et Biophysica Acta*.

[117] Gianturco SH, Ramprasad MP, Lin AHY, Song R, Bradley WA (1994). Cellular binding site and membrane binding proteins for triglyceride-rich lipoproteins in human monocyte-macrophages and THP-1 monocytic cells. *Journal of Lipid Research*.

[118] Gianturco SH, Brown SA, Via DP, Bradley WA (1986). The *β*-VLDL receptor pathway of murine P388D1 macrophages. *Journal of Lipid Research*.

[119] Elsegood CL, Pal S, Roach PD, Mamo JCL (2001). Binding and uptake of chylomicron remnants by primary and THP-1 human monocyte-derived macrophages: determination of binding proteins. *Clinical Science*.

[120] Elsegood CL, Mamo JCL (2006). An investigation by electron microscopy of chylomicron remnant uptake by human monocyte-derived macrophages. *Atherosclerosis*.

[121] Cohn JS (2008). Are we ready for a prospective study to investigate the role of chylomicrons in cardiovascular disease?. *Atherosclerosis Supplements*.

[122] Mangat R, Su JW, Lambert JE (2011). Increased risk of cardiovascular disease in Type 1 diabetes: arterial exposure to remnant lipoproteins leads to enhanced deposition of cholesterol and binding to glycated extracellular matrix proteoglycans. *Diabetic Medicine*.

[123] Phillips C, Murugasu G, Owens D, Collins P, Johnson A, Tomkin GH (2000). Improved metabolic control reduces the number of postprandial apolipoprotein B-48-containing particles in type 2 diabetes. *Atherosclerosis*.

[124] Curtin A, Deegan P, Owens D, Collins P, Johnson A, Tomkin GH (1996). Elevated triglyceride-rich lipoproteins in diabetes. *Acta Diabetologica*.

[125] Mero N, Syvänne M, Taskinen M-R (1998). Postprandial lipid metabolism in diabetes. *Atherosclerosis*.

[126] Mero N, Malmström R, Steiner G, Taskinen MR, Syvänne M (2000). Postprandial metabolism of apolipoprotein B-48- and B-100-containing particles in type 2 diabetes mellitus: relations to angiographically verified severity of coronary artery disease. *Atherosclerosis*.

